# Calcium-Induced Activity and Folding of a Repeat in Toxin Lipase from Antarctic *Pseudomonas fluorescens* Strain AMS8

**DOI:** 10.3390/toxins12010027

**Published:** 2020-01-01

**Authors:** Nur Shidaa Mohd Ali, Abu Bakar Salleh, Raja Noor Zaliha Raja Abd Rahman, Thean Chor Leow, Mohd Shukuri Mohamad Ali

**Affiliations:** 1 Enzyme and Microbial Technology Research Center, Faculty of Biotechnology and Biomolecular Sciences, Universiti Putra Malaysia, 43400 Serdang, Selangor, Malaysia; nur_shidaa@yahoo.com (N.S.M.A.); abubakar@upm.edu.my (A.B.S.); rnzaliha@upm.edu.my (R.N.Z.R.A.R.); adamleow@upm.edu.my (T.C.L.); 2 Department of Biochemistry, Faculty of Biotechnology and Biomolecular Sciences, Universiti Putra Malaysia, 43400 Serdang, Selangor, Malaysia; 3 Department of Microbiology, Faculty of Biotechnology and Biomolecular Sciences, Universiti Putra Malaysia, 43400 Serdang, Selangor, Malaysia; 4 Department of Cell and Molecular Biology, Faculty of Biotechnology and Biomolecular Sciences, Universiti Putra Malaysia, 43400 Serdang, Selangor, Malaysia

**Keywords:** RTX lipase, AMS8 lipase, family I.3, RTX parallel *β*-roll motif repeat, Ca^2+^ ion, calcium binding, folding, activity

## Abstract

It is hypothesized that the Ca^2+^ ions were involved in the activity, folding and stabilization of many protein structures. Many of these proteins contain repeat in toxin (RTX) motifs. AMS8 lipase from Antarctic *Pseudomonas fluorescens* strain AMS8 was found to have three RTX motifs. So, this research aimed to examine the influence of Ca^2+^ ion towards the activity and folding of AMS8 lipase through various biophysical characterizations. The results showed that CaCl_2_ increased lipase activity. The far-UV circular dichroism (CD) and Fourier-transform infrared (FTIR) analysis suggested that the secondary structure content was improved with the addition of CaCl_2_. Fluorescence spectroscopy analysis showed that the presence of CaCl_2_ increased protein folding and compactness. Dynamic light scattering (DLS) analysis suggested that AMS8 lipase became aggregated at a high concentration of CaCl_2_.The binding constant (K_d_) value from the isothermal titration calorimetry (ITC) analysis proved that the Ca^2+^ ion was tightly bound to the AMS8 lipase. In conclusion, Ca^2+^ ions play crucial roles in the activity and folding of the AMS8 lipase. Calcium binding to RTX nonapeptide repeats sequences will induced the formation and folding of the RTX parallel *β*-roll motif repeat structure.

## 1. Introduction

Repeat in toxin (RTX) proteins represent a broad and diverse family of proteins produced by Gram-negative bacteria. RTX proteins can be divided into several classes including RTX lipases, RTX proteases, RTX cytotoxins and Multifunctional-autoprocessing repeats-in-toxin (MARTX) [1]. RTX proteins exhibit two common characteristics. The first characteristic is the appearance of nonapeptide sequences (GGXGXDXUX) (consisting of glycine (G), aspartate (D), any amino acid (X) and a hydrophobic amino acid (U)) at the carboxy terminal. The nonapeptide sequences are responsible for creating specific calcium-binding positions and after calcium binding, the parallel *β*-roll motif repeat structure of the RTX was formed [2]. The RTX parallel *β*-roll motif repeat structure comprised nonapeptide sequences and Ca^2+^ ions [3]. The second characteristic is the style of secretion through the type I secretion system (TISS). The RTX parallel *β*-roll motif repeat structure was involved in the process of exporting the passenger protein (including RTX proteins) out of the bacterial cell through T1SS [4].

Of note, the RTX parallel *β*-roll motif repeat structure has been hypothesized to require Ca^2+^ ion to exert its biological function and influence in the folding and stabilization of many protein structures [3]. To date, the exact function of the RTX parallel *β*-roll motif repeat structure is still poorly understood [1]. So far, it is not understood how the Ca^2+^ ion induces the folding of the RTX parallel *β*-roll motif repeat structure and how it helps to sustain the structural integrity of the whole protein structure. The cases may be linked to the activity, folding and secretion of RTX proteins [5]. However, previous studies have suggested that the RTX parallel *β*-roll motif repeat structure is responsible for internal chaperones, enhancers of the secretion process and receptor-binding domains [4].

In 2013, a new RTX lipase from *Pseudomonas fluorescence* (AMS8 lipase) belonging to the I.3 subfamily was isolated from Antarctic soil (psychrophilic bacteria). The crude enzyme showed maximum activity at 20 °C [6]. A previous study applied a computational approach (homology modeling) to predict the 3D structure of AMS8 lipase [7]. AMS8 lipase consists of 476 amino acids. The predicted structure of AMS8 lipase obtained from Ali et al. (Supplementary Materials Figure S1) [7] consisted of the catalytic (residue 1–392) and non-catalytic (residue 393–405) domains. The catalytic domain at the N-terminal was rich in *α*-helices (*α* 20) and the non-catalytic domain was monopolized by *β*-strands (*β* 17). The catalytic domain consisted of an *α*/*β* hydrolase fold and catalytic triad, which combined Ser^207^, Asp^255^ and His^313^ residues. Commonly, lipase can occur in two conformational categories: active and inactive. The active or inactive conformation states were determined based on the lid conformation, either open or closed. For AMS8 lipase, there are two lid structures covering the catalytic site on the catalytic domain [8].

Based on the predicted structure, AMS8 lipase contains one Zn^2+^ and six Ca^2+^ ions. Besides, the predicted model of AMS8 lipase also showed the presence of nonapeptide sequences at the C-terminal. The RTX nonapeptide repeats sequences will form the RTX parallel *β*-roll motif repeat structure specifically upon binding with Ca^2+^ ions [9]. AMS8 lipase consists of three RTX parallel *β*-roll motif repeat structures (residue 373–405). These motifs constituted a specific type of Ca^2+^ ion-binding site that was necessary for the formation of the parallel *β*-roll motif repeat structure. Ca^2+^ ions were crucial in the formation of the RTX parallel *β*-roll repeat structure and the presence of Ca^2+^ ions is also important for both the folding and stability of many protein domains [10]. Besides, metal ions can be a factor in the stabilization and functionality of the protein structure [11].

In the current work, RTX lipase (AMS8 lipase) from *Pseudomonas fluorescens* strain AMS8 (accession numbers ADM87309) has been studied. Previous homology modeling revealed that the AMS8 lipase predicted structure consists of Ca^2+^ ions that maybe involved in the functionality/folding of the RTX parallel *β*-roll motif repeat structure and also the whole AMS8 lipase structure. Thus, this research aimed to examine the influence of Ca^2+^ ions in the activity and folding of AMS8 lipase. To further characterize the functionality/folding differences influenced by the binding of Ca^2+^ ions towards AMS8 lipase, the physicochemical characteristic of AMS8 lipase was investigated by utilizing biophysical approaches.

## 2. Results and Discussion

### 2.1. Calcium-Binding Site and RTX β-Roll Motif Repeat Structure of the AMS8 Lipase Predicted Model

**Figure 1:**
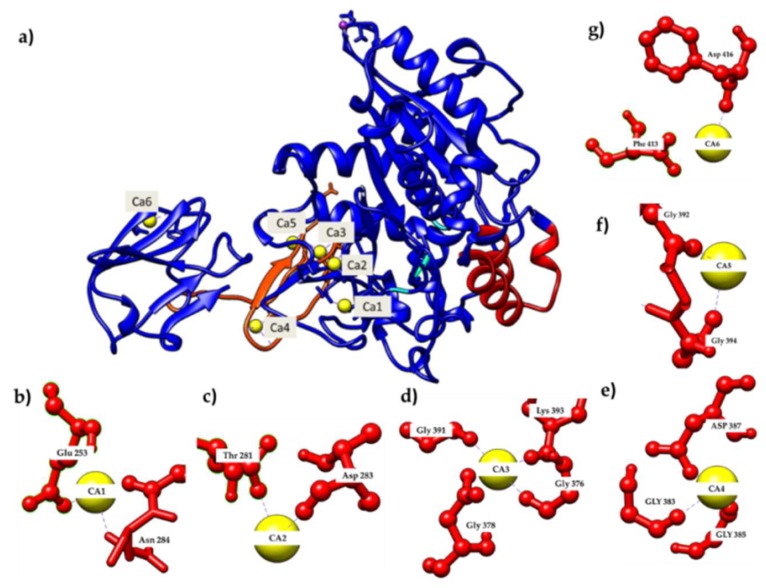
The predicted model of AMS8 lipase obtained from Ali et al. showed the presence of Ca^2+^ ions [7]. (**a**) Calcium-binding site of the AMS8 lipase predicted structure. (**b**) The Ca1 was bound with Asn^284^ and Glu^253^ residues, (**c**) Ca2 was bound with Asp^283^ and Thr^281^ residues, (**d**) Ca3 was bound with Asp^378^, Gly^391^, Lys^393^ and Gly^376^ residues, (**e**) Ca4 was bound with Asp^387^, Gly^383^ and Gly^385^ residues, (**f**) Ca5 was bound with Gly^392^ and Gly^394^ residues, and (**g**) Ca6 was bound with Phe^413^ and Asp^416^ residues.

**Figure 2:**
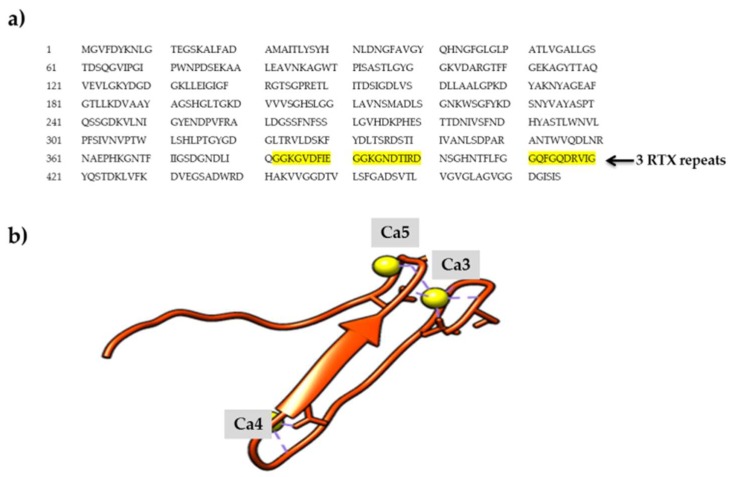
The repeat in toxin (RTX) parallel *β*-roll motif repeat structure of AMS8 lipase. (**a**) The AMS8 lipase sequence contains three RTX parallel *β*-roll motif repeat structures (GGXGXDXUX) at the C-terminal (residue 373–405) as highlighted in yellow. (**b**) Ca3, Ca4, and Ca5 were bound to the RTX parallel *β*-roll motif repeat structures.

In order to further analyze the presence of Ca^2+^ ions in the AMS8 lipase predicted structure, we used the database to explore the calcium-binding site of each of the Ca^2+^ ions. The ligplot of the interaction involving the calcium-binding site of analysis from the pictorial database of 3D structures in the Protein Data Bank (PDBsum) was visualized using YASARA software [12] Figure 1 shows that the Ca1 was bound with Asn^284^ (2.294 Å) and Glu^253^ (2.209 Å) (Figure 1b), Ca2 with Asp^283^ (2.328 Å) and Thr^281^ (2.381 Å) (Figure 1c), Ca3 with Asp^378^ (2.236 Å), Gly^391^ (2.227 Å), Lys^393^ (2.237 Å) and Gly^376^ (2.272 Å) (Figure 1d), Ca4 with Asp^387^ (2.270 Å), Gly^383^ (2.341 Å) and Gly^385^ (2.310 Å) (Figure 1e), Ca5 with Gly^392^ (2.344 Å) and Gly^394^ (2.339 Å) (Figure 1f) and Ca6 with Phe^413^ (2.178 Å) and Asp^416^ (1.523 Å) (Figure 1g). Both Ca1 and Ca2 were located at the catalytic domain, while Ca3, Ca4 and Ca5 were located at the RTX parallel *β*-roll motif repeat structure in the non-catalytic domain and Ca6 was also located at the non-catalytic domain of the AMS8 lipase structure. Figure 2 shows the Ca^2+^ ion involved in the formation of the RTX parallel *β*-roll motif repeat structure.

The demand for Ca^2+^ ions in the RTX parallel *β*-roll motif repeat structure formation was reported for *Escherichia coli* a-hemolysin [9] and *Bordetella pertussis* CyaA [13]. The binding of Ca^2+^ ions to the RTX parallel *β*-roll motif repeat structure only occurs during secretion (outside the bacterial cell), since the concentration of Ca^2+^ ions inside the bacterial cell was low (0–100 nM) [14]. This condition makes the RTX parallel *β-*roll motif repeat structure remain floppy/unfolded before being secreted out of the bacterial cell through the TISS [15,16]. Calcium binding to the RTX parallel *β*-roll motif repeat structure in the C-terminal will assist the functional/folding of secreted RTX proteins outside the bacterial cell [17,18,19]. Besides, the presence of Ca^2+^ ions is also compatible with both the stability and folding of many protein domains [10].

Binding and release of Ca^2+^ ions change the structural properties of the involved calcium-binding proteins such that they will switch their state during interaction with other proteins or during enzymatic reaction [20]. The parts of the consecutive turns and *β*-strands form a right-hand parallel *β*-helix that is stabilized by the binding of Ca^2+^ ions. Meanwhile, without Ca^2+^ ions, these RTX parallel *β*-roll motif repeat structures appear to be mostly disordered [3,21].

### 2.2. Effect of Different Ca^2+^ Ion Concentrations on AMS8 Lipase Activity

The AMS8 lipase inclusion body (IB) was harvested from bacterial cultures through centrifugation. AMS8 lipase IBs were solubilized under denaturing conditions using urea to remove the Ca^2+^ ions. The previous study proved the workability of urea-based microbial carbonate precipitation as a Ca^2+^ ion removal technology [22]. Twelve different CaCl_2_ concentrations (0, 5, 10, 20, 30, 40, 50, 60, 70, 80, 90 and 100 mM) had been used to optimize the best concentration of CaCl_2_ needed by the unfolded AMS8 lipase to correctly refold to its native structure.

A clear lysate was obtained during the refolding process without any protein precipitation as all the debris was removed during the solubilization process [6]. The refolded protein was assayed and analyzed to Sodium Dodecyl Sulfate-Polyacrylamide Gel Electrophoresis (SDS-PAGE) analysis. It was assumed that the AMS8 lipase had already been purified in the solubilization and refolding process since SDS-PAGE detected only the AMS8 lipase band. A single band of protein in the expected size (65 kDa) was observed after the refolding process with different CaCl_2_ concentrations. The appearance of CaCl_s_ had a significant influence on the lipase activity (U/mL) of AMS8 lipase. The effect of Ca^2+^ ions on refolded lipase activity is presented in Table 1. The positive enzymatic activity by refolded AMS8 lipase indicates the successful refolding process since the protein was active.

Nevertheless, the total lipase activity depended on the concentration of CaCl_2_ used. With increasing CaCl_2_ concentrations (5–70 mM), the lipase activity was progressively accelerated and reached the highest lipase activity in the presence of 80 mM CaCl_2_, in contrast to the negative control (0 mM). CaCl_2_ also improved the lipase activity from diverse microorganisms such as *Bacillus subtilis* 168, *Bacillus thermoleovorans* ID-1 and*Pseudomonas aeruginosa* EF2. As previously published, higher concentrations of CaCl_2_ resulted in greater lipase activity of cold-adapted lipase from *Pseudomonas fragi* [23].

However, a further increase in CaCl_2_ concentration (90–100 mM) led to a decrease in the lipase activity since the value started to decrease from approximately 262.5 to 226.7 U/mL. These conditions may be caused by the aggregation of the protein which will be discussed in dynamic light scattering (DLS) analysis. Based on the lipase activity results, it was proven that CaCl_2_ plays a vital function in the protein refolding process and catalytic activity.80 mM CaCl_2_ was the optimum concentration needed for the refolding process of AMS8 lipase, in which the highest lipase activity had been detected.

### 2.3. Circular Dichroism (CD) Analysis

**Figure 3:**
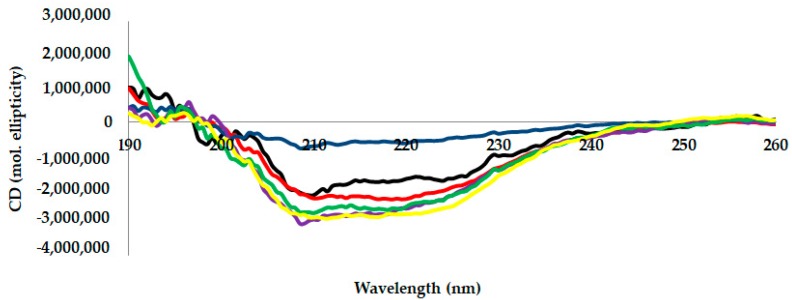
Far-UV CD spectra of AMS8 lipase in the presence of different CaCl_2_ concentrations (0 mM: black line, 20 mM: red line, 40 mM: blue line, 60 mM: purple line, 80 mM: green line and 100 mM: yellow line). The protein concentrations and enzyme solutions were maintained at 1 mg/mL in 1 mM Tris–HCl buffer in the presence of different CaCl_2_ concentrations.

Circular dichroism measurements are tools generally used to measure a protein’s secondary structure [24]. Figure 3 shows the far-UV CD spectra (observation range: 190–260 nm) of AMS8 lipase with different CaCl_2_ concentrations. Five different CaCl_2_ concentrations (0 (control), 20, 40, 60, 80 and 100 mM) were used to perform this analysis. Far-UV CD spectra revealed that the presence of CaCl_2_ improved the secondary structure of AMS8 lipase. The far-UV CD spectrum (Figure 3) gives a shift of the negative peak to approximately 218 nm and the presence of a positive peak at approximately 190 nm in the presence of CaCl_2_. This spectrum is indicative of an enhanced *β*-sheet secondary structure and is similar to the data published by Bauche et al. [25].

The *β*-sheet data value increased in the presence of CaCl_2_ (20–60 mM). In a detailed observation, the percentage of *β*-sheet content obtained (20–60 mM) was not very different in three different CaCl_2_ concentrations. Nevertheless, the value started to decrease when higher CaCl_2_ concentrations were added (90–100 mM). The decrease in lipase activity was caused by the decrease in*β*-sheet content, as shown in Table 2. This condition may be due to the aggregation of protein as reported by DLS analysis.

Based on the overall results, the presence of 80 mM CaCl_2_ gave the best secondary structure content, since the percentage of *β*-sheet and α-helix was high compared to the other concentrations. Referring to the model built previously, the catalytic domain of AMS8 lipase was mainly built by an *α*/*β* fold and this domain is suggested to be a fundamental domain for the catalytic efficacy of AMS8 lipase [6]. The helical structure of AMS8 is located inside the catalytic domain. Thus, this domain is proposed to be liable for the activity and stability of the protein despite the flexibility properties of the non-catalytic domain which represents a role in the structure and function of the protein [6].

### 2.4. Fourier-Transform Infrared Spectroscopy (FTIR) Analysis

FTIR spectroscopy is another biophysical tool that can also provide data about the secondary and tertiary structure content of the proteins by shining infrared radiation on a sample [26]. Interestingly, FTIR spectroscopy can also be used to analyze the calcium binding of protein [27]. Interaction of the protein with metal ions resulted in significant vibration and spectroscopy signals which was detected at wavelengths corresponding to three groups, namely, Amide A at 3500 cm^−1^ (NH), Amide I at 1700–1600 cm^−1^ (C=O) and Amide II at 1550 cm^−1^ (C–N and N–H) [28]. Based on the FTIR analysis, two peaks were detected at the Amide 1 and Amide A wavelength. Both peaks present were sensitive to secondary structure content and metal ion interaction (Ca^2+^ ion). For the interaction of Ca^2+^ ion binding, the Amide I peak (1700–1600 cm^−1^) and the Amide A peak (near 3500 cm^−1^) were slightly lower at low Ca^2+^ ion concentrations (0–60 mM). However, the spectral intensity of the bands mentioned above was observed to increase significantly at a higher Ca^2+^ ion concentration (80–100 mM). The increase in the spectral intensity was due to the reaction of the Ca^2+^ ion with the Amide I (C=O), Amide II (C–N, N–H) and Amide A (NH) regions of the protein.

**Figure 4:**
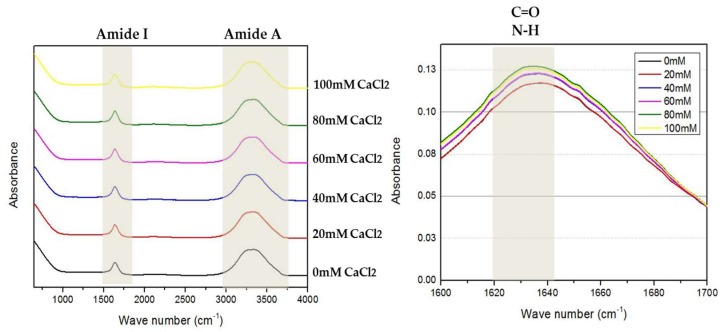
Fourier-transform infrared spectroscopy (FTIR) analysis in the presence of different CaCl_2_ concentrations (0 mM: black line, 20 mM: red line, 40 mM: blue line, 60 mM: purple line, 80 mM: green line and 100 mM: yellow line). The wavelength at 1600–1700 cm^−1^ represents Amide I and the wavelength at 1621–1640 cm^−1^ represents *β*-sheet content. The protein concentrations and enzyme solutions were maintained at 1 mg/mL in 1 mM Tris–HCl buffer.

The FTIR spectrum was asymmetric, with a shoulder between 1600 and 1700 cm^−1^, proposing the appearance of absorption bands in this wavelength. The wave number of the maximum absorption of AMS8 lipase in the presence of CaCl_2_ was 1627 cm^−1^, which was assigned to *β*-sheet content. The Amide I wavelength precisely at 1621–1640 cm^−1^ represents *β*-sheet content (Figure 4). Based on the graph, the absorbance value shows the intensity of the stretching vibrations of the C=O and N–H bonds. From here, we can see the increment of *β*-sheet content in AMS8 lipase without the presence of CaCl_2_ (0 mM) and with the presence of CaCl_2_ (20, 40, 60, 80 and 100 mM). AMS8 lipase with 80 mM CaCl_2_ gave the highest intensity compared to other concentrations used. FTIR analysis for the *β*-sheet content of AMS8 lipase was correlated to the CD analysis (Table 2). Both showed that the percentage of *β*-sheet content increased in the presence of CaCl_2_, and 80 mM gave the highest *β*-sheet content. As with the CD analysis, the percentage of AMS8 lipase *β*-sheet content in the presence of 100 mM decreased.

### 2.5. Fluorescence Spectroscopy Analysis

**Figure 5:**
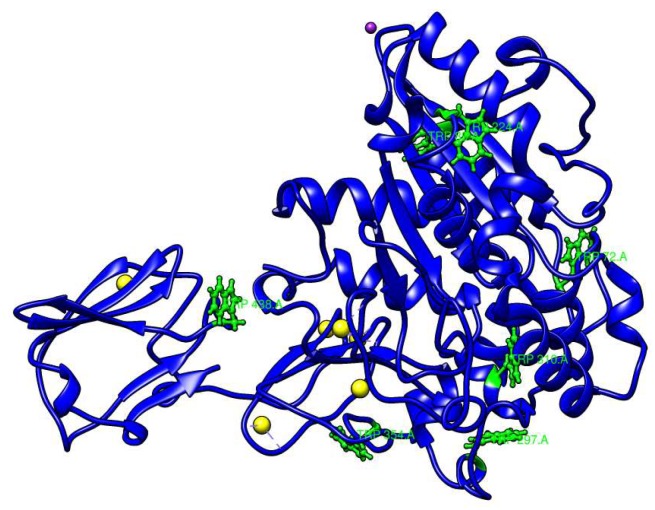
Distribution of tryptophan residues in the AMS8 lipase predicted structure. There are seven tryptophan residues at positions 72, 89, 224, 297, 310, 354 and 438 (green color).

**Figure 6:**
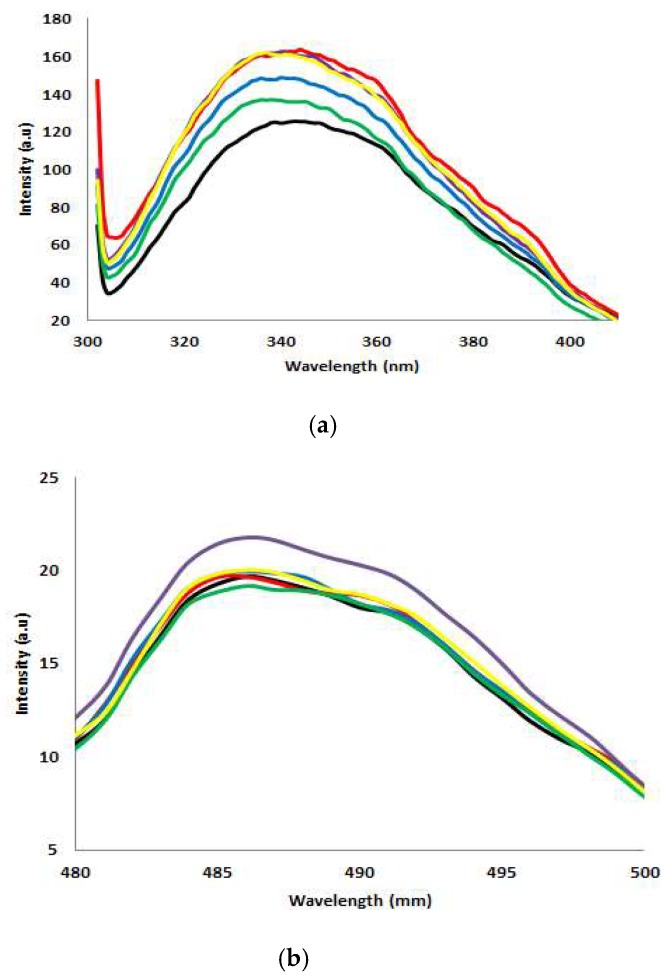
Fluorescence spectroscopy analysis. (**a**) Intrinsic fluorescent analysis of AMS8 lipase with different CaCl_2_ concentrations (0 mM: black line, 20 mM: red line, 40 mM: blue line, 60 mM: purple line, 80 mM: green line and 100 mM: yellow line). (**b**) Extrinsic fluorescent analysis of AMS8 lipase with different CaCl_2_ concentrations (0 mM: black line, 20 mM: red line, 40 mM: blue line, 60 mM: purple line, 80 mM: green line and 100 mM: yellow line).

AMS8 lipases contain seven Trp residues at positions 72, 89, 224, 297, 310, 354 and 438 (Figure 5). Figure 6 shows the folding/compactness analysis of AMS8 lipase with different CaCl_2_ tested using fluorescence spectroscopy (intrinsic and extrinsic ANS fluorescence). The fluorescence of lipases is due to aromatic residues (Trp, Tyr and Phe). Nevertheless, Trp residue is the most sensitive when exposed to fluorescence compared to Tyr and Phe residue. Intrinsic fluorescence parameters such as intensity and peak wavelength (λ_max_) provide excellent means to study structural dynamics and lipase polarity [29]. The conformational changes in the fluorescence intensity of the AMS8 lipase with different CaCl_2_ concentrations reflect an alteration in the solvent accessibility and tertiary structure of aromatic residues.

As shown in Figure 6a, the AMS8 lipase without the addition of CaCl_2_ as a control (0 mM) had maximum emission of 340 nm, and the addition of different CaCl_2_ concentrations (20, 40, 60, 80 and 100 mM) impact the fluorescence intensity. The fluorescence intensity of the AMS8 lipase increased slightly in the presence of CaCl_2_. AMS8 lipase was found to be blue shifted since the intensity decreased at 340 nm. The blue shift in the λ_max_ present reflected the changes in the Trp microenvironment and its accessibility to an aqueous solution. It showed that the Trp was shielded from bulk water but not placed in the hydrophobic core of the lipases. Without the CaCl_2_, the λ_max_ decreased and reached a minimum value of 304 nm. The structural propensity changes in the protein in response to CaCl_2_ indicate the involvement of specific intra-molecular interactions in the protein structure, which confers extra stability. In some ways, the appearance of metal ions may alter the electrostatics of the protein. The fluorescence of the protein is dependent on the environment and the folding of the protein. The more compact structure of the protein leads to a higher fluorescence intensity than the disordered/denatured counterpart [30]. Thus, the increased fluorescence intensity of AMS8 lipase in the presence of Ca^2+^ ion was reasonably contributing to the structural compactness of the protein in the aqueous medium.

The extrinsic ANS fluorescence further supported the effect of different CaCl_2_ concentrations. ANS binding upon the hydrophobic group of protein was also used for the detection of the formation of the molten globule state of the protein [31]. Extrinsic ANS fluorescence spectra of AMS8 lipases with 80 mM of CaCl_2_ gave the highest intensity at 486 nm (Figure 6b). It shows the Trp residues were exposed to the environment/solvent. However, the AMS8 lipase peak emission intensity of another different CaCl_2_ concentration (20, 40, 60 and 100 mM) was significantly decreased. Therefore, the decrease in ANS fluorescence intensity was significantly linked to the reduced exposure (Trp buried inside the hydrophobic core) of Trp residue to the environment/solvent.

### 2.6. Dynamic Light Scattering (DLS) Analysis

The DLS technique was applied to measure the size of the protein (other particles) by scattered light intensity. This method has recently been used in the study of folding and denaturation, aggregation, and also the complex formation of proteins in solution [24]. The DLS data was read based on the differences between the aggregation size of the sample and control. AMS8 lipase with various CaCl_2_ concentrations (20, 40, 60, 80 and 100 mM) had been used as a sample and AMS8 lipase without being treated with CaCl_2_ (0 mM) had been used as a control.

**Figure 7:**
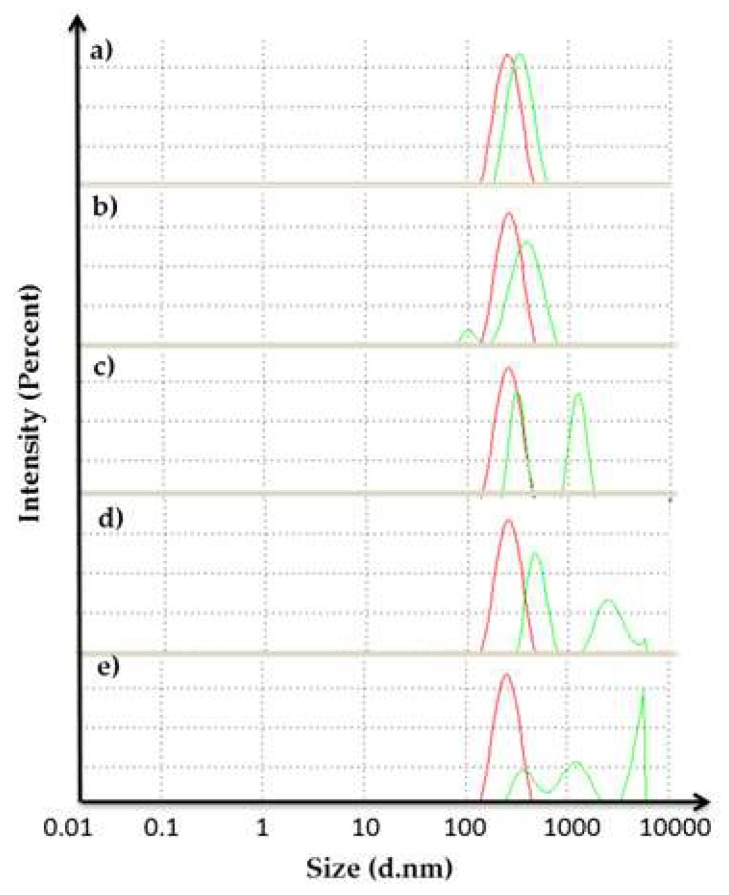
Dynamic light scattering (DLS) graph for the enhancement stage of the reaction (size in diameter (d.nm)). Concentration of CaCl_2_ in AMS8 lipase: (**a**) 20; (**b**) 40; (**c**) 60; (**d**) 80 and (**e**) 100 mM. The red curve represents the control and the green curve represents the sample.

Based on the result in Figure 7, increasing the CaCl_2_ concentration influenced the size of the protein. The average size of particles (in diameter) of 20, 40, 60, 80 and 100 mM CaCl_2_ was increased from 245.5 to 302.2, 319.6, 697.2, 863.0 and 1223 nm respectively. When the size of the protein was huge, the PI value would also be high because the sample was too polydispersed. Based on the quality report from the software, the sample might aggregate and contain large particles. This condition may lead to decreasing lipase activity at a higher concentration of CaCl_2_ as discussed in Section 2.2.

### 2.7. Isothermal Titration Calorimetry (ITC) Analysis

In order to define the binding affinity of the AMS8 lipase with calcium, the enzyme–calcium interaction was observed using ITC. ITC was used to study all sorts of binding reactions, including protein–ligand reactions. ITC is the alternative method to calculate the thermodynamic parameters associated with the structural transformation of a single molecule or the non-covalent interaction of several molecules [32].

Representative calorimetric titrations of AMS8 lipase with CaCl_2_ were performed at 25 °C. Injections were performed repeatedly and resulted in peaks that became smaller as the biomolecule became saturated after 25 injections (200s injection interval) had been performed. The normalized results are displayed under each titration result. The AMS8 lipase reaction with CaCl_2_ was exothermic, since a negative peak was observed. For this experiment, 5 µM of AMS8 lipase and 50 µM of CaCl_2_ were used. The concentration used was different from the other biophysical tools, since ITC only required a small amount of sample concentration. ITC was among the most sensitive biophysical tools that only required a small concentration of sample and ligand compared to other biophysical tools that had been used in this study.

Based on ITC analysis, we are focusing on the binding affinity between the protein and ligand. The binding affinity (dissociation constant (K_d_)) was used to measure the strengths of biomolecular interactions. The K_d_ value for AMS8 lipase with the ligand was 1.458 × 10^−7^ M. Since the K_d_ value observed was small, it proved that the Ca^2+^ ion was tightly bound to the AMS8 lipase. The lower the value of the K_d_, the higher the ligand’s binding affinity to AMS8 lipase. If the K_d_ value was high, the ligand would be weakly bound to the protein (other target molecules) [33].

## 3. Conclusions

In conclusion, AMS8 lipase from *Pseudomonas fluorescence* belongs to the RTX lipases group since it contains an RTX parallel *β*-roll motif repeat structure at the C-terminal. This lipase contains three RTX parallel *β*-roll motif repeat structures and six Ca^2+^ ions. We have proven that in the presence of Ca^2+^ ions, the lipase activity of AMS8 lipase increased. This shows that the contribution of calcium binding performed a vital function in sustaining the structural integrity of AMS8 lipase. The evidence from lipase activity was further analyzed by using various biophysical characterizations. The biophysical characterization analyses (Far-UV CD spectra, FTIR spectroscopy, fluorescence spectroscopy, DLS and ITC) revealed that the presence of Ca^2+^ ions improved the secondary structure and made the folding of AMS8 lipase better compared to untreated AMS8 lipase (without Ca^2+^ ion). The result from various biophysical characterizations of the role of the calcium-induced activity/folding of RTX-containing lipase AMS8 may contribute fresh insights toward the calcium-binding relationship of the RTX lipases study.

## 4. Materials and Methods

### 4.1. Calcium-Binding Site of AMS8 Lipase Predicted Model

A previous study adopted a homology modelling approach to predict the AMS8 lipase structure [6]. The predicted 3D model (GenBank accession No: ADM87309.1) was obtained from Ali et al. [6] and was used in this research to analyze the calcium-binding site and RTX *β*-roll motif repeat structure. The ligplot of the interaction involving the calcium-binding site of AMS8 lipase was analyzed using the pictorial database of 3D structures in the Protein Data Bank (PDBsum) and visualized by using YASARA software [34].

### 4.2. Expression, Solubilization and Refolding of AMS8 IBs with Different Ca^2+^ Ion Concentrations

The expression of AMS8 lipase was conducted as mentioned by Jalil et al. with slight modifications [12]. The *E. coli* strain BL21(DE3) with recombinant plasmid pET32b/*lipAMS8* was induced with IPTG (0.1 mM) for AMS8 lipase production at 20 °C with shaking at 180× *g* for 18 h. The production of AMS8 lipase was qualified by streaking the culture on an agar plate containing tributyrin 1% (*v*/*v*) and ampicillin (50 µg/mL). After three days of incubation at 4 °C, the hydrolysis of tributyrin by a lipase was proven by the development of a halo zone (around colony) on the plate.

After the expression process, the pellet was collected and treated with 10 mL Tris–HCl buffer (50 mM) containing urea (8 M) at pH 8.0 for solubilization for 3 h (4 °C) with constant agitation. The solubilized AMS8 lipase was centrifuged for 30 min (10,000× *g*), and the supernatant containing IBs was refolded via a 10× dilution method by using the peristaltic pump with the slowest flow rate (0.5 mL/min) against Tris–HCl (50 mM) at pH 8.0 in various CaCl_2_ concentrations (0, 5, 10, 20, 30, 40, 50, 60, 70, 80, 90 and 100 mM). The refolded AMS8 lipase with different CaCl_2_ concentrations was assayed for lipase activity and protein content. Later, AMS8 lipase was used for SDS-PAGE and biophysical characterization analysis. The crude enzyme had been used as a positive control and AMS8 lipase without being treated with CaCl_2_ (0 mM) had been used as the negative control.

### 4.3. Determination of Lipase Activity and Protein Content

As stated by Kwon and Rhee, lipase activity was determined by the protocol [35]. The emulsion of olive oil (Bertoli, Italy) was made by the emulsification of olive oil and a buffer solution (Tris–HCl, pH 8.0) (1:1 ratio). The reaction mixture comprising 250 mL of emulsion, 990 μL Tris–HCl (pH 8.0) buffer, 20 μL of CaCl_2_ (20 mM) and 10 μL enzymes was prepared before incubation for 30 min at 20 °C with 180× *g* shaking. Afterward, 1 mL of HCl (6N) and by 5 mL of isooctane were combined to stop the reaction. The reaction mixtures were left for 30 min at 25 °C. Then, 1 mL copper pyridine solution (pH 6.1) was mixed with the upper layer reaction. The absorbance (715 nm) of lipase activity was determined using a Biochrom WPA UV/visible spectrophotometer (Cambridge, UK). The protein concentration of AMS8 lipase was determined by Bradford assay [36] using the Bradford reagent (AMRESCO, Solon, OH, USA).

### 4.4. Biophysical Characterizations

#### 4.4.1. SDS-PAGE (Sodium Dodecyl Sulfate-Polyacrylamide Gel Electrophoresis) Analysis

SDS-PAGE was conducted according to the method defined by Laemmli using 12% (*w*/*v*) of resolving gel and 4% (*w*/*v*) of stacking gels [37]. After the refolding process, the protein sample (1 mg/mL) was resuspended into 10× sample buffer (Tris–HCl (1 M, pH 6.8), 20% (*w*/*v*) SDS and 1% (*v*/*v*) bromophenol blue). The protein samples (10 µL) were electrophoresed on 40% acrylamide gel at 210 V, 60 mA for 50 min using a small electrophoresis chamber (Criterion Vertical Electrophoresis Cell, Bio-rad, Hercules, CA, USA). The gel was stained for 10 min with 0.25% Coomassie brilliant blue R 250 and destained overnight in destaining solution acetic acid:methanol:distilled water (ratio 1:1:8) [11]. The unstained protein marker (Fisher Scientific, Hampton, NH, USA) was used to estimate the molecular protein size.

#### 4.4.2. Circular Dichroism (CD) Spectra Analysis

The secondary structure analysis of the AMS8 lipase was measured using JASCO 815 spectropolarimeter (JASCO, Tokyo, Japan). The secondary structure contents of AMS8 lipase with CaCl_2_ were valued based on the far-UV (190–260 nm) using a 1 mm path cell length at 20 °C. Refolded AMS8 lipase protein (1 mg/mL) with varying CaCl_2_ concentrations (0 (control), 20, 40, 60, 80 and 100 mM) was prepared in Tris–HCl (1mM, pH 8.0) buffer. The spectral analysis of AMS8 lipase was subtracted with the subsequent blank containing Tris–HCl (1 mM, pH 8.0). All measurements were repeated three times. The secondary structure contents (beta, helix, turn and random) of AMS8 lipase were analyzed using the method by Yang et al. [38], using Spectra Manager^TM^ Suite Software (JASCO, Tokyo, Japan).

#### 4.4.3. Fourier-Transform Infrared (FTIR) Spectroscopy Analysis

The FTIR spectroscopy analysis was completed using Cole-Parmer Scientific Experts (US). FTIR was measured in Tris–HCl buffer (1 mM, pH 8.0) in the presence of various CaCl_2_ concentrations (0 (control), 20, 40, 60, 80 and 100 mM) at a spectral range of 4000–600 cm^−1^ with a nominal resolution of 2 cm^−1^ of 100 scans.

#### 4.4.4. Fluorescence Spectra Analysis

The intrinsic fluorescence spectra of the protein were measured by a Cary Eclipse Fluorescence Spectrophotometer (Agilent, Santa Clara, CA, USA) using a quartz fluorimeter cuvette (100 mm light path, 1.4 mL) at 25 °C. The protein sample (1 mg/mL) was tested at various CaCl_2_ concentrations (0 (control), 20, 40, 60, 80 and 100 mM). The excitation wavelength was 290 nm, and the emission was scanned at 300 to 400 nm. Binding of 8-anilino-1-naphthalenesulfonic acid (ANS) to the protein was analyzed by measuring the fluorescence of ANS at 25 °C. The protein sample (1 mg/mL) and ANS (50 µM) were dissolved in Tris–HCl buffer (80 mM, pH 8.0). The mixture was tested at various CaCl_2_ concentrations (0 (control), 20, 40, 60, 80, and 100 mM). The protein sample with ANS was scanned at a wavelength of 380 nm for excitation and 390 to 650 nm for emission.

#### 4.4.5. Dynamic Light Scattering (DLS) Analysis

The measurement of protein particle size in refolded AMS8 lipase with different CaCl_2_ concentrations was carried out using a Zetasizer APS DLS machine (Malvern Panalytical, Malvern, UK) at 830 nm and the results were analyzed using Zetasizer software [39]. The protein samples (1 mg/mL) were tested at various CaCl_2_ concentrations (0 (control), 20, 40, 60, 80 and 100 mM) and were used for DLS evaluation. The total volume of 150 µL of the mixture was transferred into 96-well microplates before injecting into the DLS. The DLS system was set to a temperature of 25 °C, with Tris–HCl as a buffer and latex as a standard sample throughout the analysis. The result was set to be in normal resolution.

#### 4.4.6. Isothermal Titration Calorimetry (ITC) Analysis

The protein–ligand measurements were performed using a Nano ITC, TA Instrument (US). The cell contained 300 µL of degassed AMS8 lipase (5 µM) with Tris–HCl (1 mM, pH 8.0) buffer was titrated with Tris–HCl (1 mM, pH 8.0) buffer containing CaCl_2_ (50 µM). Ligand solutions (50 µM CaCl_2_) were injected until there were no more heat changes. Titrations of the Tris–HCl (1 mM, pH 8.0) buffer with the CaCl_2_ (50 µM) were performed with the same parameters as mentioned above, and these reference data were deducted from the standard. NanoAnalyzer software was used to analyze ITC data.

## Figures and Tables

**Table 1:** The refolded AMS8 lipase activity (U/mL) been analyzed with varying CaCl_2_ concentrations.

CaCl_2_ Concentration (mM)	Lipase Activity (U/mL)
0	26.40 ± 0.74
5	137.99 ± 7.70
10	157.08 ± 1.81
20	160.00 ± 3.69
30	169.23 ± 2.92
40	173.88 ± 2.98
50	183.67 ± 0.93
60	198.48 ± 1.58
70	218.23 ± 0.25
80	262.49 ± 3.91
90	255.18 ± 1.74
100	226.69 ± 1.32

The data are shown as the mean ± S.D. (error bars) of the lipase activity (U/mL) of AMS8 lipase with different CaCl_2_ concentrations, performed in triplicate (*n* = 3). Significant statistical differences according to ANOVA (*p* < 0.05).

**Table 2:** The changes in AMS8 lipase secondary structure contents analyzed with varying CaCl_2_ concentrations.

CaCl_2_ Concentrations (mM)	Percentage (%)			
	Beta	Helix	Turn	Random
0	7.7	25.3	30.7	36.5
20	12.9	29.7	22.1	35.2
40	22.1	19.4	21.7	36.8
60	24.3	22.3	21.2	36.3
80	26.7	21.2	19.9	32.2
100	19.6	24.7	20.3	35.4

## Supplementary Materials

The following are available online at https://www.mdpi.com/2072-6651/12/1/27/s1, Figure S1: The predicted structure of AMS8 lipase obtained from Ali et al. (2013).
