# Supplementary material for: Calcium-Induced Activity and Folding of a Repeat in Toxin Lipase from Antarctic Pseudomonas fluorescens Strain AMS8
Source: Toxins (Basel). 2020 Jan 1;12(1):27. doi: 10.3390/toxins12010027 (PMC7020413; doi:10.3390/toxins12010027)
Supplement: Supplementary file 1 [file toxins-12-00027-s001.pdf]

Calcium-Induced Activity and Folding of a Repeat in Toxin Lipase from Antarctic *Pseudomonas fluorescens* Strain AMS8

Nur Shidaa Mohd Ali [1,2], Abu Bakar Salleh [1], Raja Noor Zaliha Raja Abd Rahman [1,3], Thean Chor Leow [1,4] and Mohd Shukuri Mohamad Ali [1,2,*]

[1] Enzyme and Microbial Technology Research Center, Faculty of Biotechnology and Biomolecular Sciences, Universiti Putra Malaysia, 43400 Serdang, Selangor, Malaysia; nur_shidaa@yahoo.com (N.S.M.A.); abubakar@upm.edu.my (A.B.S.); rnzaliha@upm.edu.my (R.N.Z.R.A.R.); adamleow@upm.edu.my (T.C.L.)

[2] Department of Biochemistry, Faculty of Biotechnology and Biomolecular Sciences, Universiti Putra Malaysia, 43400 Serdang, Selangor, Malaysia.

[3] Department of Microbiology, Faculty of Biotechnology and Biomolecular Sciences, Universiti Putra Malaysia, 43400 Serdang, Selangor, Malaysia.

[4] Department of Cell and Molecular Biology, Faculty of Biotechnology and Biomolecular Sciences, Universiti Putra Malaysia, 43400 Serdang, Selangor, Malaysia.

* Correspondence: mshukuri@upm.edu.my; Tel.: +603-9769 6721

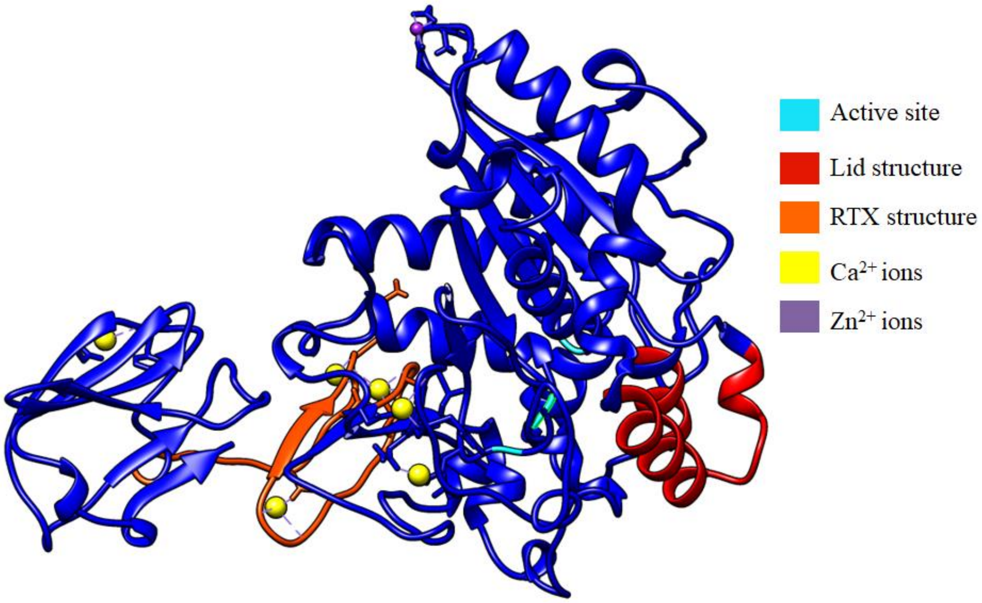

Figure S1: The predicted structure of AMS8 lipase obtained from Ali et al. (2013).
